# Changes in Microbiota Across Developmental Stages of *Aedes koreicus*, an Invasive Mosquito Vector in Europe: Indications for Microbiota-Based Control Strategies

**DOI:** 10.3389/fmicb.2019.02832

**Published:** 2019-12-10

**Authors:** Niccolò Alfano, Valentina Tagliapietra, Fausta Rosso, Mattia Manica, Daniele Arnoldi, Massimo Pindo, Annapaola Rizzoli

**Affiliations:** Fondazione Edmund Mach, Research and Innovation Centre, Trento, Italy

**Keywords:** *Aedes koreicus*, microbiota, developmental stages, larval habitats, vector control, transstadial transmission, paratransgenesis, *Asaia*

## Abstract

Since it has been understood that gut microbiota of vector mosquitoes can influence their vector competence, efforts have been undertaken to develop new control strategies based on host microbiota manipulation, and aimed at suppressing the vector population or replacing it with a less competent one. For the proper design of such control strategies it is necessary to know the microbiota composition of the target vector species, how it is acquired, and how it changes throughout the host’s life cycle. In this study, 16S rRNA amplicon high-throughput sequencing was used to characterize the changes in microbiota from the aquatic environment (larval breeding sites) to the different developmental stages of field-collected *Aedes koreicus* in Italy, an emerging invasive mosquito species in Europe and a potential vector of several pathogens. The bacterial communities of the aquatic breeding sites, larvae, pupae and adults showed distinctive structures to one another. Indeed, 84% of community members were unique to a given sample type. Nevertheless, almost 40% of the sequences generated were assigned to bacteria detected in all sample types, suggesting the importance of bacteria transstadially transmitted from water to the adult stage in constituting mosquito microbiota. Among these, genus *C39* largely constituted water microbiota, family *Burkholderiaceae* was the most abundant in larvae and pupae, and genus *Asaia* dominated adult communities. In addition, *Asaia* constituted a core microbiota across all sample types. Our results suggest that the microbiota of *Ae. koreicus* mosquitoes is composed by a community which derives from the aquatic bacteria of the larval breeding sites, is then filtered by the larval gut, where only certain members are able to persist, rearranged by metamorphosis and finally modified by the change in diet at the adult stage. Understanding how the microbiota of *Ae. koreicus* changes through the mosquito life cycle represents a first step in selecting bacterial candidates for use in microbiota-based intervention measures for this species. The properties which *Asaia* exhibits in this species, such as dominance, high prevalence and transstadial transmission, prevent the use of *Wolbachia* but make *Asaia* an ideal candidate for paratransgenesis.

## Introduction

The spread of vector-borne diseases, caused by factors such as climate and land-use changes, urbanization, globalized trade and travel, which together increase the risk of the introduction of vectors to new environments, represents a considerable public health concern regarding new outbreaks worldwide (Suk and Semenza, 2014; Early et al., 2016). One of the most important groups of pathogen-transmitting vectors is mosquitoes, which include several species well-known for vectoring *Plasmodium* parasites that cause malaria in humans (*Anopheles* spp.) and arboviruses such as dengue, yellow fever, Zika, chikungunya and West Nile (*Aedes* and *Culex* spp.). During the last decade, invasive mosquitoes, together with the pathogens they carry, especially arboviruses, have considerably expanded their geographic range and caused an increasing number of human outbreaks in Europe (Barzon, 2018; Semenza and Suk, 2018).

*Aedes koreicus* is native to Japan, China, South Korea, and eastern Russia (Tanaka et al., 1979), but in the last decade has been spreading throughout Europe. Since its first detection in Belgium in 2008 (Versteirt et al., 2013), it has been recorded in Italy in 2011 (Capelli et al., 2011), Russia, Switzerland and Slovenia in 2013 (Bezzhonova et al., 2014; Suter et al., 2015; Kalan et al., 2017), Germany in 2015 (Werner et al., 2016) and Hungary in 2016 (Kurucz et al., 2016). The species has successfully established local populations in Belgium and north-eastern Italy (Montarsi et al., 2013; Versteirt et al., 2013) confirming its ability to colonize new areas in temperate regions thanks to its higher tolerance to cold temperatures compared to its main competitor species, *Aedes albopictus*. In particular, in Italy, after its first detection in 2011 in the province of Belluno (north-eastern Italy) (Capelli et al., 2011), the species has spread rapidly south-westerly over the last few years (Montarsi et al., 2015b) being reported in the province of Trento in 2013 and since then at increasing abundances in all the main valleys of the province (unpublished data collected during ordinary mosquito surveillance activities). Furthermore, *Ae. koreicus* has been recently recorded in Liguria region (north-western Italy) (Ballardini et al., 2019), not only revealing the potential to invade northern Italy in the next decade (Montarsi et al., 2015b), but also extending the risk of spread throughout Europe (Montarsi et al., 2013; Marcantonio et al., 2016). The expansion of *Ae. koreicus* across Europe may represent a serious threat to human and animal public health in function of the potential role of the species as a vector of several infectious diseases. Indeed, the species is purported to be associated with the transmission of the Japanese encephalitis virus (Miles, 1964; Shestakov and Mikheeva, 1966) and has been shown to be able to transmit the chikungunya virus in the laboratory (Ciocchetta et al., 2018). Furthermore, it has been proven to transmit the parasitic nematodes *Dirofilaria immitis* to dogs (Montarsi et al., 2015a) and *Brugia malayi* to humans (KCDC, 2007).

Application of control measures is necessary to prevent the further expansion of the species or, at least, to limit the potential spread of the pathogens it may vector. To this purpose, novel intervention strategies based on manipulation of mosquito-associated microbiota, which has the potential to modulate host vector competence (Dennison et al., 2014; Hegde et al., 2015), have been developed (van Tol and Dimopoulos, 2016; Saldaña et al., 2017). These include the introduction into vector populations of: (i) entomopathogenic bacteria to increase mosquito mortality (Huang et al., 2017); (ii) the endosymbiotic bacterium *Wolbachia*, which can induce sterilization in the host (Huang et al., 2017) and lower its vector competence, when artificially transferred into a naive vector (Moreira et al., 2009; Hoffmann et al., 2011; Ferguson, 2018); (iii) vector symbionts which have been genetically engineered to secrete anti-pathogen molecules (paratransgenesis) (Wang and Jacobs-Lorena, 2013).

Microbial-based intervention strategies need to be species-specific, and the design of future control measures depends on knowing the microbiota composition of each target vector species, how it is acquired and changes throughout its life cycle. This is particularly relevant for mosquitoes, which undergo a complete metamorphosis shifting from an aquatic to a terrestrial life style and may acquire their microbiota at several stages throughout their lifespan. To date, the microbiota of *Ae. koreicus* has been analyzed only at the adult stage (Rosso et al., 2018). In addition, since the mosquito microbiota is highly variable, as a consequence of different factors such as environment and individual history (Wang et al., 2011; Gimonneau et al., 2014; Muturi et al., 2017, 2018b; Bascuñán et al., 2018; Romoli and Gendrin, 2018), it is important to discriminate opportunistic bacteria from a core microbiome, which is composed of bacteria stably associated with a certain mosquito species and developmental stages (Shade and Handelsman, 2012).

In this study, we used 16S rRNA amplicon high-throughput sequencing to characterize the microbiota associated with each developmental stage of *Ae. koreicus* and its larval habitats in an area (province of Trento, Italy) where the species is rapidly expanding. The aim of this study was to understand how the microbiota of *Ae. koreicus* changes during its development, which bacteria are acquired from the environment, which are transstadially transmitted, and define the core microbiota within and between life stages. These data represent a first step in identifying potential bacterial targets useful to develop control strategies tailored to this species.

## Materials and Methods

### Study Sites and Sample Collection

*Aedes koreicus* has been recorded in the province of Trento, north-eastern Italy, since 2013, after its spread from the neighboring province of Belluno, and is now well established in this area (Montarsi et al., 2013, 2015b). Three sampling sites were selected based on the known presence of *Ae. koreicus* population in this territory (Supplementary Figure 1): “Tezze” site (Lat: 45.991377; Long: 11.671797; 251 m a.s.l) was located in the cemetery of Tezze village (municipality of Grigno); “Villa Agnedo” site (Lat: 46.0486667; Long: 11.5408333; 383 m a.s.l) was located in a forested area in Villa Agnedo village (municipality of Castel Ivano); “Strigno” site (Lat: 46.066721976; Long: 11.52514577; 492 m a.s.l) was located in a private property in Strigno village (municipality of Castel Ivano) (Supplementary Figure 1). Samples were collected in July and August 2017, a period when mosquito populations reach peak abundances. Water samples and preimaginal stages (larvae and pupae) were collected simultaneously at all three sampling sites, from a catch basin in Tezze and Strigno and from a concrete bin in Villa Agnedo. Both superficial and deeper (5–10 cm depth) water samples were collected at each site. Superficial water samples were collected by placing four Isopore Membrane Filters (diameter 47 mm, pore size 0.2 μm) (Merck Millipore Ltd.) onto the aquatic surface layer at each site using sterile tweezers and waiting for 20 s. For deeper water samples, 60 mL of water at 5–10 cm depth were collected by submerging a sterilized glass bottle (100 mL capacity) and filtered in two portions (30 mL at a time) using a Millipore Sterile filtration system (Merck Millipore Ltd.). One superficial and two deeper water samples were collected per site, except for Site 3 where two superficial and four deeper water samples were taken, for a total of 12 water samples. Larvae at fourth instar and pupae were collected with a dipper and immediately transferred in a 50 mL tube with a pasteur pipette and transported alive to the laboratory. Adult mosquitoes (males and females) were collected by a modified leaves aspirator along drywalls in the proximity of the breeding sites and transported alive to the laboratory. Ten samples per site for each mosquito developmental stage (larvae, pupae, adult males and adult females) were collected, making a total of 120.

Larvae and pupae were placed in 70% ethanol and morphologically identified at species level (Montarsi et al., 2013; Ciocchetta et al., 2017). The species of each pupal sample was confirmed as *Ae. koreicus* using a PCR targeting the ND4 gene (Cameron et al., 2010). Adult males and females were killed by placing them at −20°C for 5 min, and then morphologically identified at species level (Montarsi et al., 2013). All samples were surface sterilized with 70% ethanol and rinsed with water for molecular analysis. Midgut extraction was performed using sterile tweezers and entomological pins on a sterilized slide with a 100 μL drop of 1x PBS under a stereomicroscope (80x magnification) frequently cleaned with ethanol during dissection.

### DNA Extraction

DNA extraction of water samples was performed using the Dneasy PowerWater kit (Qiagen) following the manufacturer’s protocol. For each site, the four membrane filters from the surface water were pooled in a DNA bead tube and extracted together, while the two filters obtained from the deep water were extracted individually. DNA from midgut samples of larvae, pupae and adults was extracted using the QIAmp DNA Investigator kit (Qiagen) after incubating overnight in ATL buffer and Proteinase K at 56°C and shaking gently (Thermo-shacker Grant Bio). Final elution volume was done in 25 μL of ATE buffer and DNA was quantified with the Qubit 2.0 Fluorometer (Invitrogen). To control for contamination of bacteria introduced during the DNA extraction, a negative control consisting of a blank sample was included for each batch of extractions. Since no quantifiable extract was produced from each negative control, they were not further processed.

### Polymerase Chain Reaction

The specific primers 341 F (5′-CCTACGGGNGGCWGCAG-3′) and 805Rmod (5′-GACTACNVGGGTWTCTAATCC-3′) (Herlemann et al., 2011; Klindworth et al., 2013) (with degenerate bases suggested by Apprill et al., 2015) with overhang Illumina adapters were used to generate amplicons (∼460 bp) covering the hypervariable V3 and V4 regions of the 16S rRNA gene. All PCRs were conducted in 25 μL of volume and prepared under sterile conditions. Each PCR reaction contained 2.5 μL of 10 × Fast Start High Fidelity Reaction Buffer (Roche), 0.5 μL of 10 mM dNTP mix (Fermentas), 1 μL of 10 μM forward and reverse primers, 0.25 μL of 5 U/μL Fast Start High Fidelity Enzyme blend (Roche), 15 ng of DNA and sterile water to volume. Reactions without template served as negative controls. All PCR amplifications were carried out using a Veriti-96 Well Thermal Cycler (Applied Biosystem) under the following cycling conditions: initial denaturation at 94°C for 3 min, 35 cycles of 15 s denaturation at 94°C, 45 s annealing at 55°C and 1 min and 10 s elongation at 72°C, followed by a final 8 min elongation step at 72°C.

### Amplicon Library Preparation and Sequencing

The PCR products were checked on 1.5% agarose gel and cleaned from free primers and primer dimers using the Agencourt AMPure XP system (Beckman Coulter) following the manufacturer’s instructions. Subsequently, dual indices and Illumina sequencing adapters Nextera XT Index Primer (Illumina) were attached by 7 cycles PCR (16S Metagenomic Sequencing Library Preparation, Illumina). Since PCR negative controls gave weak amplification products, they were pooled, converted into libraries and sequenced in order to control for possible contaminations. After purification by the Agencourt AMPure XP system (Beckman Coulter), the final libraries were analyzed on a Tapestation 2200 platform (Agilent Technologies) and quantified using the Quant-IT PicoGreen dsDNA assay kit (Thermo Fisher Scientific) by the Synergy2 microplate reader (Biotek). Finally all the libraries were pooled in an equimolar way in a final amplicon library and analyzed on a Tapestation 2200 platform (Agilent Technologies). Barcoded libraries were sequenced on an llumina MiSeq (PE300) platform.

### Bioinformatics and Statistical Analysis

A total of 16,068,979 paired-end sequence reads 300 bp long were generated (average = 121,735 paired-end reads per sample, standard deviation [*SD*] = 33,071) (Supplementary Table 1), sorted by index sequences and imported into QIIME2 (version 2018.4)^1^ using the q2-demux plugin. Sequences were quality filtered, trimmed, de-noised and merged using DADA2 (Callahan et al., 2016) as implemented in the q2-dada2 plugin to infer exact amplicon sequence variants (ASVs), which were then summarized in a feature table. ASVs are observational units resolved down to the level of single-nucleotide differences over the sequenced gene region. Denoising methods inferring ASVs allows for a higher resolution and more accurate estimates of diversity and composition than common methods using clustering steps to generate OTUs at a certain similarity percentage (Callahan et al., 2017). Chimeric sequences were identified and removed via the consensus method in DADA2. Singletons were removed to minimize the effect of spurious sequences. Taxonomy was assigned to the ASVs using a naive Bayes feature classifier trained on the SILVA 99% OTU database (release 132)^2^ (Quast et al., 2013) trimmed to include the V3-V4 region of the 16S rRNA gene (q2-feature-classifier plugin). Organellar (mitochondrial and chloroplast) and other contaminating (unassigned, unidentified, Eukaryotes, Archaea) sequences were filtered out from the dataset. The ASVs counts were corrected with the reads from the negative controls. All ASVs found in the negative control samples were removed from the ASV table unless the count in a real sample was >10 times higher than the mean ASV count in the negative controls (Dickson et al., 2017). After these filtering steps, overall 7,693 ASVs and 6,922,302 sequences were retained (Supplementary Table 1). Taxonomy summaries with relative abundance data at different taxonomic levels were generated from the ASV table where ASVs with an abundance <0.01% of the total read count were removed. The representative sequences were aligned with MAFFT (Katoh and Standley, 2013) and a phylogenetic tree was generated from the masked alignment using FastTree (Price et al., 2010). Alpha (within-sample diversity) and beta-diversity (between-sample diversity) analyses were performed with the q2-diversity plugin at a rarefied sampling depth of 20,194. Rarefaction resulted in the exclusion from diversity analyses of nine samples (three males, three females, and three pupae samples) that had < 20,194 sequences per sample. Four alpha diversity metrics were computed. Observed OTUs is an estimator of species richness. Faith’s Phylogenetic Diversity (PD) is a measure of biodiversity that considers phylogenetic difference between species. Pielou’s evenness measure how equally a community is numerically distributed among the species. Shannon index takes into account both abundance and evenness of species present in a community. We tested for significant effects of categorical metadata (sample type and site) for alpha diversity using both a pairwise and an “all-group” Kruskal–Wallis test and report a Benjamini and Hochberg corrected *p*-value (“*q*-value”) (Benjamini and Hochberg, 1995). Beta diversity was estimated by calculating the Bray–Curtis (Bray and Curtis, 1957) dissimilarity between samples. Beta diversity analysis using Non-Metric Multidimensional Scaling (NMDS, R: vegan: metaMDS) based on Bray-Curtis dissimilarity distance matrix was carried out to visualize differences between microbial communities in samples from different sample types or sites. Furthermore, we performed a permutational multivariate analysis of variance (PERMANOVA, R: vegan: Adonis) with 10,000 permutations to test the influence of categorical metadata (sample type and site) on the differences in microbial composition between samples measured by Bray-Curtis distance.

Differential abundance analysis between pairs of sample types (water-larva; larva-pupa; pupa-adult; males-females) was performed using ANalysis of Composition Of Microbiomes (ANCOM) (Mandal et al., 2015) and balance trees (Morton et al., 2017) as implemented with the q2-composition and q2-gneiss QIIME2 plugins, respectively. ANCOM is applied to identify features that are differentially abundant (i.e., present in different abundances) across sample groups. Balances are used to infer meaningful partitions of microbes that can explain the differences between sample groups. Both methods are helpful to overcome the issue of compositionality of microbiome data and have been recently demonstrated to reduce false discovery rate compared to other alternatives (Weiss et al., 2017), but both are sensitive to sparsity (i.e., tables with high proportion of zero counts). To reduce the impact of sparse features, balances were inferred from ASV tables where ASVs present in only a single sample and with less than 10 sequences were filtered out. Since ANCOM assumes that few (less than about 25%) of the features are changing between groups, ANCOM was performed on ASV tables where only ASVs that were prevalent in at least 25% of the samples from the two sample types compared were retained and then collapsed at the genus level. The abundances of the bacterial genera and ASVs identified by ANCOM and balances as differentially abundant between sample types were inspected in order to prevent the presence of sparse taxa from giving false-positive results.

The core microbiota was calculated using the q2-feature-table plugin in QIIME2 as the group of ASVs with at least 10 reads in at least 50% of the samples from each sample type.

The rarefied ASV table was manipulated to extract unique and shared ASVs among sample types and to obtain the most abundant ASVs among the shared ASVs for each sample type using two scripts modified from Bascuñán et al. (2018). The resulting tables were used respectively to generate the pie charts showing the percentage of reads and ASVs that were unique or shared between sample types, and the boxplots showing the percentage of reads of the shared ASVs with maximal abundances per sample type.

Venn diagrams showing either the overlap between the core microbiota of each sample type or the number of shared and unique ASVs between sample types were generated using the web tool available at http://bioinformatics.psb.ugent.be/webtools/Venn/.

### Taxonomic Analysis of a Dominant *Acetobacteraceae* ASV

One single ASV, which was initially assigned to the genus *Acetobacter* (family *Acetobacteraceae*), was found dominant in adult samples and also present as core ASV in all sample types. Since the assignment was obtained at low confidence (0.76 with a threshold value of 0.7), we decided to sequence a longer fragment of the 16S rRNA gene in order to be more confident in the taxonomic assignment of this ASV. *Acetobacteraceae*-specific primers were used to amplify a 1432 bp long 16S rRNA gene fragment, as previously described (Yukphan et al., 2004). The PCRs were performed on two female adult samples where over 96% of the Illumina sequencing reads were assigned to the *Acetobacter* ASV in order to avoid interference with other bacterial sequences. We checked that this longer sequence had 100% identity to the representative sequence of the ASV previously assigned to the genus *Acetobacter*. When the longer sequence was subjected to homology search using MegaBlast against the NCBI’s nucleotide (nt) database, the first match was found with an *Asaia* species at 97.3% identity. The first match with the *Acetobacter* genus was at 96% identity. We also imported this longer sequence into QIIME2 and classified it using a naive Bayes classifier pre-trained on the SILVA 99% OTU full-length sequences database using the q2-feature-classifier plugin. The sequence was assigned to the genus *Asaia* with confidence 0.81. Finally, the sequence was aligned with 142 accessions (about 1500 bp long) of *Acetobacter* and *Asaia* spp. from the non-redundant Silva Ref 16S rRNA gene database (release 132)^3^ (Quast et al., 2013) and a single outgroup (*Gluconobacter cerinus*, AB024492) using MAFFT version 8 (Katoh and Standley, 2013). Phylogenetic analysis was performed using the maximum-likelihood method based on the general time reversible substitution model (Lanave et al., 1984) available in RAxML version 3 (Stamatakis, 2014), including 500 bootstrap replicates to determine the node support. The sequence from this study clustered together with other *Asaia* sequences with good node support (Supplementary Figure 2). Based on these results, we assigned the ASV to the genus *Asaia*.

## Results

### Diversity Analyses

The rarefaction curves reached saturation for all sample types indicating that our sampling effort has captured most of the bacterial communities diversity (Supplementary Figure 3), except for the water samples where the high bacterial diversity observed was partially uncovered.

**FIGURE 1:**
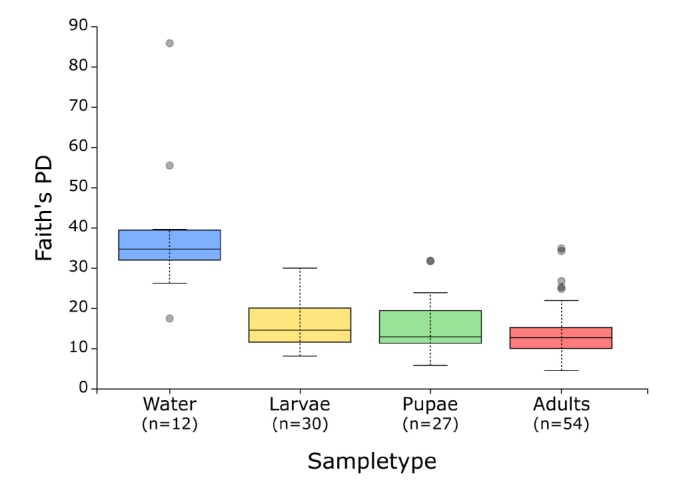
Faith’s Phylogenetic Diversity (PD) for each sample type. Faith’s PD was calculated at a rarefaction depth of 20,194 sequences/sample. Boxes represent the interquartile range (IQR) between the first and third quartiles (25th and 75th percentiles, respectively), and the horizontal line inside the box defines the median. Whiskers represent the lowest and highest values within 1.5 times the IQR from the first and third quartiles, respectively. Circle symbols indicate outliers (values greater than 1.5 times and less than three times the IQR).

ASVs richness was significantly different among sample types (Kruskal–Wallis test: *H* = 26.8 with *p* < 0.0001 for Observed OTUs; *H* = 31.1 with *p* < 0.0001 for Faith’s PD): in particular, it was higher in the water samples than in the mosquito samples (Figure 1, Supplementary Table 2, and Supplementary Figure 4). Since no statistical differences in alpha diversity metrics were found between deep water and superficial water (Supplementary Table 3), we merged the two datasets and show them as a single sample type “water” (Supplementary Table 2). We also found that pupal communities were significantly more evenly distributed than other sample types (Kruskal–Wallis test: *H* = 22.4 with *p* < 0.0001 for Pielou’s evenness; *H* = 20.4 with *p* < 0.001 for Shannon) (Supplementary Table 2).

**FIGURE 2:**
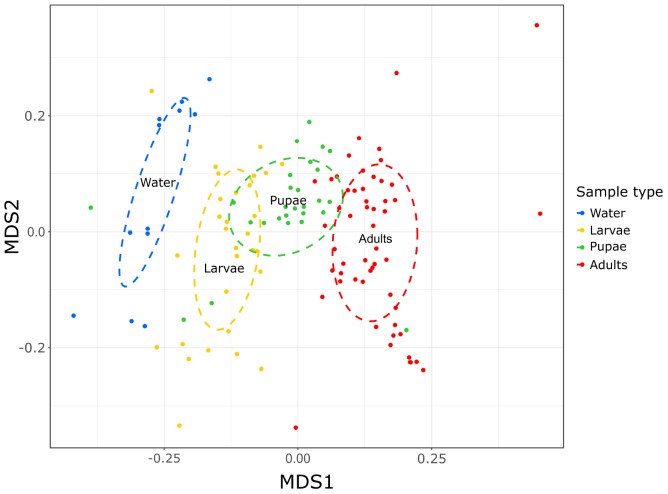
Different structure of the bacterial communities of the larval breeding sites and developmental stages of *Aedes koreicus*. Bacterial community structure is represented by non-metric multidimensional scaling (NMDS) ordination of Bray–Curtis distances.

To determine whether the structure of bacterial communities differed between sample types, we performed a NMDS analysis on the Bray-Curtis dissimilarity matrix generated on the basis of the ASVs abundance. Each sample type clustered separately from one another (Figure 2; stress = 0.21) and PERMANOVA confirmed that bacterial communities differed significantly based on sample types (*F* = 6.9; 10,000 permutations; *R*^2^ = 0.15; *p* < 0.0001). When analyzed separately, deep and superficial water samples clustered together (Supplementary Figure 5) and PERMANOVA found no significant differences between them (*F* = 0.9; 10,000 permutations; *R*^2^ = 0.08; *p* = 0.3). Therefore, we presented here the two merged datasets as “water” (Figure 2). Interestingly, by observing the NMDS plots, it can be seen that water, larvae, pupae and adults, even if clustering apart from one another, are distributed along a continuum from water to larvae to pupae to adults, with water and adults at the two ends (Figure 2). Therefore, water samples show higher similarity with larval than with pupal or adult samples, and adults are more similar to pupae than to the other sample types.

We further found that the same differences based on sample type could be observed within each sampling site (*F* = 3.9; 10,000 permutations; *R*^2^ = 0.15; *p* < 0.001). However, the sampling site showed a less pronounced effect (*F* = 3.7; 10,000 permutations; *R*^2^ = 0.05; *p* < 0.0001), with no clear clustering pattern (Supplementary Figure 6).

### Microbiota Taxonomical Composition

At the phylum level, the bacterial communities of both water and mosquito samples were dominated by *Proteobacteria* with abundances ranging from 66% in larvae to 84% in adults. *Proteobacteria* were represented mainly by the *Gammaproteobacteria* class, with abundances around 60% in water, larvae and pupae, whereas in adult communities we observed a sharp decrease in this class (30%) in favor of *Alphaproteobacteria* (53%) (Supplementary Figure 7 and Supplementary Table 4). Also *Bacteroidetes* and *Actinobacteria*, which were main components of aquatic, larval and pupal communities (ranges 7–15% and 4–8%, respectively) were detected at lower frequencies in adults (5 and 2%). *Firmicutes* were almost absent in water (<1%), but were observed at higher abundances in all mosquito developmental stages (4% in larvae, 5% in both pupae and adults).

**FIGURE 3:**
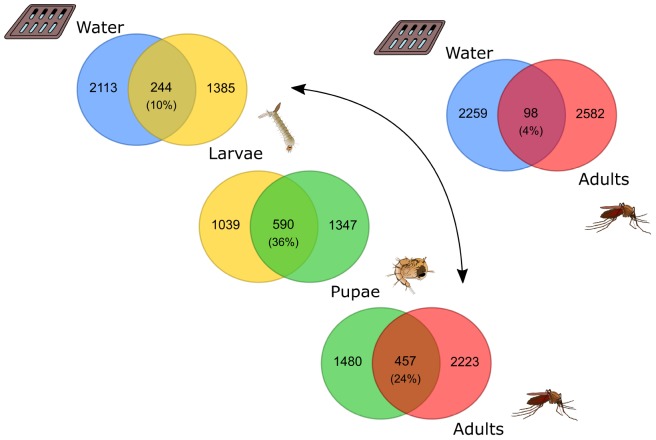
Limited overlap of bacterial communities between sample types. Venn diagrams showing the numbers of shared or unique bacterial ASVs among pairs of sample types. The venn diagrams were generated from the rarefied ASV table.

**FIGURE 4:**
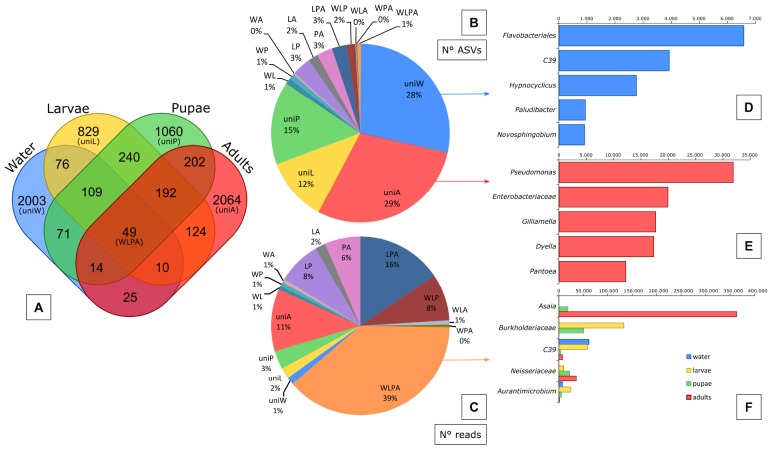
Abundance and taxonomic composition of the microbiota shared or unique to each sample type. Venn diagram showing the number of bacterial ASVs unique to each sample type (‘uni’) or shared among each combination of sample types (W, water; L, larvae; P, pupae; A, adults) **(A)**. Pie charts illustrating the percentage of ASVs **(B)** and reads **(C)** that were unique or shared between sample types. Taxonomic composition (5 most abundant genera) of the ASVs unique to water **(D)**, unique to adults **(E)** or shared among all sample types (WLPA) **(F)**. The identification at higher levels (family or order) is reported for unidentified genera. The venn diagrams and the charts were generated from the rarefied ASV table.

**FIGURE 5:**
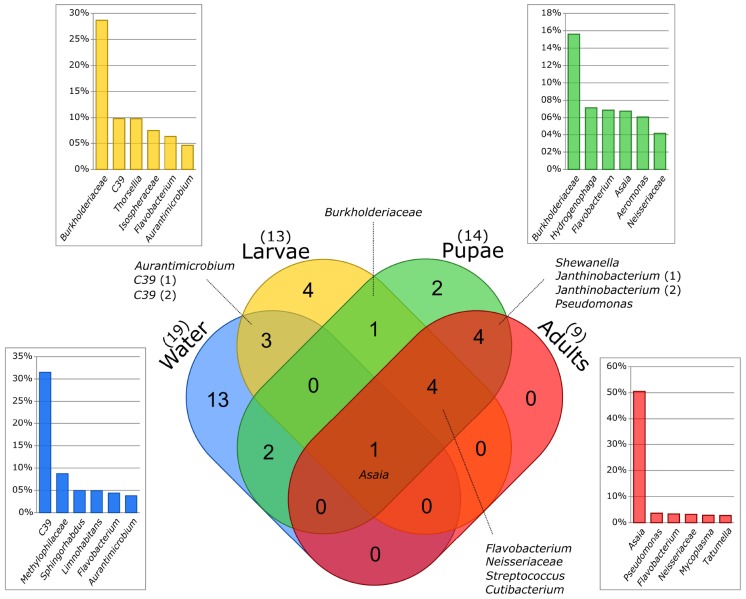
Overlap between the core microbiome of each sample type. The core microbiome is represented by the group of ASVs with at least 10 reads in at least 50% of the samples from each sample type. The taxonomic identification of the core ASVs shared among sample types is reported. In addition, the relative frequencies of the 6 most abundant genera of the bacterial community of each sample type are reported. The identification at the family level is reported for unidentified genera.

At a finer taxonomic resolution we observed stronger variations across sample types. Even though some community members were shared, a considerable proportion was unique to each sample type: only 10% of the bacteria identified in water were recovered in larvae; 36% of the bacteria found in larvae were present also in pupae and 24% of the bacteria inhabiting the pupae guts were detected in the adults (Figure 3). Finally, only 4% of the ASVs identified in the aquatic habitats were observed in the adult guts (Figure 3). We observed indeed that among all the identified ASVs, 84% of them was unique to a certain sample type. In particular, water (28%) and adults (29%) had most of the unique ASVs (Figures 4A,B). Most of the ASVs unique to adults were assigned to the genera *Pseudomonas*, *Gilliamella*, *Dyella* and *Pantoea*, and to the family *Enterobacteriaceae* (Figure 4D), while most of the ASVs unique to water belonged to the order *Flavobacteriales* and to the genera *C39*, *Hypnocyclicus* and *Paludibacter* (Figure 4E). The ASVs shared across different combinations of sample types represented together only the 16% of the total number of ASVs (Figure 4B). In particular, the ASVs shared across all the sample types analyzed (water, larvae, pupae and adults = WLPA) represented a minimal fraction (1%) (Figure 4B). Nevertheless, these shared ASVs were the most abundant in term of reads, with the WLPA ASVs representing 39% of total sequences, and those shared among mosquitoes developmental stages (larvae, pupae and adults = LPA) 16% (Figure 4C). WLPA ASVs, in particular, constituted a considerable portion of the bacterial communities of each sample type, accounting for 49% of total sequences of water samples, 42% of larval samples, 30% of pupal samples and 39% of adult samples. The ASVs unique to a certain sample type, instead, had very low abundances representing only 1–3% of the total sequences, except water’s unique ASVs which accounted for 11% of the total sequences (Figure 4C). Looking at the taxonomic profile of the WLPA ASVs, we observed a shift in dominance from *Gammaproteobacteria* in water, larvae and pupae to *Alphaproteobacteria* in adults (Supplementary Figure 8), similar to what observed at the whole community level (Supplementary Figure 7 and Supplementary Table 4). At a finer level, the majority of the WLPA sequences were assigned to the genera *Asaia*, *C39* and *Aurantimicrobium*, and to the families *Burkholderiaceae* and *Neisseriaceae* (Figure 4F). A WLPA ASV belonging to the genus *Asaia* was part of the core microbiotas of each sample type (Figure 5). This was the most abundant ASV accounting alone for 22% of the total number of sequences, and was detected in 73% of the samples. The *Asaia* genus, almost completely represented by this ASV, dominated adults communities with 50% abundance, but was detected at 7% in pupae and at negligible frequency (<1%) in water and larvae (Figures 4F, 5). Larval and pupal communities were dominated by an ASV associated to the *Burkholderiaceae* family (29 and 16%, respectively), which was also part of the core microbiotas of both sample types (Figures 4F, 5). This family was detected at low frequencies in water (2%) and in adults (<1%). Two ASVs assigned to the genus *C39* and one to the genus *Aurantimicrobium* were part of the core microbiotas of both water and larvae (Figure 5). Indeed, *C39* dominated the water microbiota (31%) and constituted 10% of larval sequences, while *Aurantimicrobium* represented about 4% of both communities (Figures 4F, 5). Both were still detected in pupal and adult stages, but below 1%. Finally, an ASV belonging to the family *Neisseriaceae*, despite not reaching high frequencies (<4% in all sample types), was detected in 79% of either larval, pupal or adult samples and was part of the core microbiota of each mosquito developmental stage (Figures 4F, 5).

Interestingly, when we classified the WLPA ASVs based on in which sample type they reached their maximal abundance, we observed that they were represented at higher frequency in the contiguous sample type than in others (Supplementary Figure 9). For example, those ASVs which were maximally abundant in adult samples were also present in the samples from the previous developmental stage, i.e., pupae (Supplementary Figure 9).

Differential abundance analysis revealed that in the transition from water to larval bacterial communities there was a significant decrease in the proportion of the *C39* genus and a simultaneous increase in the *Burkholderiaceae* family. From larvae to pupae there was a significant loss of the genus *Aurantimicrobium*. Finally, the differences among the pupal and adult microbiotas were driven by different ratios of the genus *Asaia*, which increased considerably in abundance in the adults, and of the family *Burkholderiaceae* and genus *Hydrogenophaga*, which were at higher frequencies in pupae (Supplementary Figure 10 and Supplementary Table 5).

### Differences Between Adult Male and Female Microbiota

Females and males showed no statistical differences in alpha diversity metrics (Supplementary Table 3) and also similar bacterial community structure: they formed overlapping clusters in the NMDS plots (Supplementary Figure 5) and no significant differences were found by PERMANOVA (*F* = 0.14; 10,000 permutations; *R*^2^ = 0.002; *p* = 0.8). The gut microbiota of the two mosquito sexes showed also similar taxonomic profiles. Both communities were dominated by the phylum Proteobacteria (84 and 83%, in males and females respectively), which was mostly represented by the genus *Asaia* (47 and 53%), followed by *Pseudomonas* (4 and 3%) and *Flavobacterium* (3 and 4%) (Supplementary Figure 11 and Supplementary Table 6). Neither ANCOM nor balances identified any bacterial group which was differentially abundant between male and female mosquitoes.

## Discussion

In this study we used 16S rRNA amplicon high-throughput sequencing to characterize the dynamics of bacterial community structure from the larval breeding sites to the different developmental stages of field-collected *Aedes koreicus*, an emerging invasive mosquito species in Europe and a potential vector of several pathogens. Our results suggest that acquisition of bacteria from water and then transstadial transmission play key roles in shaping the microbiota of *Ae. koreicus*, although multiple factors likely influence and rearrange its composition throughout mosquito development.

First of all, our data revealed that the microbiota of larval breeding sites and of each of the mosquito developmental stages show distinctive community structures (Figure 2), which is in accordance with previous studies on other mosquito species (Wang et al., 2011; Dada et al., 2014; Gimonneau et al., 2014; Duguma et al., 2015; Dickson et al., 2017; Bascuñán et al., 2018). Nevertheless, the dissimilarity between water and mosquito microbial communities increased during the mosquito life cycle, as the mosquito develops from immature to adult stages (Figure 2). This difference could indicate that mosquito microbiota originates from the bacteria acquired during the aquatic immature life stages, besides those transmitted vertically from parents, but gradually diverge from that through mosquito development till the adult phase.

### From Water to Larvae: Mosquito Gut Is a Selective Environment

In accordance with the differentiation of the microbiota structure across sample types, we observed that only a small percentage of ASVs was shared among sample types, with most being unique to each sample type. In particular, the fact that 85% of water ASVs were found exclusively in the aquatic habitat, while only 10% was recovered in larvae (Figure 3), indicates that the vast majority of bacteria present in water were not able to colonize the larval gut. Our results are in agreement with previous observations of low numbers of shared OTUs both between water and larval samples of *Aedes japonicus* and *Aedes triseriatus* (Guégan et al., 2018) and between water and adult samples of *Aedes aegypti* (Dada et al., 2014), and seem to corroborate the idea that the gut of mosquito larvae is a selective environment where only few aquatic bacteria are able to survive (Wang et al., 2011; Gimonneau et al., 2014; Bascuñán et al., 2018; Guégan et al., 2018). This observation is based on the particular conditions bacteria have to face in this environment, such as redox potential, pH, immune responses, lytic enzymes and competition with other microorganisms or maternally inherited bacteria, which may prevent the survival of certain bacteria and foster the establishment of others (Bascuñán et al., 2018; Guégan et al., 2018).

Consistently, we found a significant drop in microbial community diversity from the aquatic environment to all mosquito developmental stages (Figure 1 and Supplementary Figure 4). This finding is in accordance with the generally low complexity of bacterial communities characterizing mosquito midguts (Minard et al., 2013) and with the studies on *Aedes* spp. reporting that water breeding sites show higher OTU richness than both larvae and adults (Dada et al., 2014; Dickson et al., 2017; Wang et al., 2018). Among the bacteria which were exclusively found in the larval breeding sites, we mainly recovered genera which are usually associated with aquatic environments: *C39* has been mainly detected in freshwater bodies (Zhou et al., 2017; Eck et al., 2019), *Hypnocyclicus* has been isolated from a hydrothermal vent (Roalkvam et al., 2015), *Paludibacter* from flooded rice paddy fields (Qiu et al., 2014) and *Novosphingobium* is associated with a variety of habitats, including the rhizosphere, lakes and seawater (Kampfer et al., 2015) (Figure 4D).

### Transmission of Some of the Larval Gut Colonizers to Subsequent Mosquito Stages

Among the bacteria ingested by larvae from water, some disappear or survive in small numbers, while others may find the right conditions to proliferate and play an important role in constituting the mosquito microbiota. In our study, a minimal portion (1%, 49 ASVs in total) of the total bacterial ASVs were identified both in water, larvae, pupae and adults. These shared ASVs presumably represented the only few bacteria which were acquired by larvae from the aquatic breeding sites and were able not only to colonize larval gut, but also to persist across developmental stages till reaching and colonizing the adult gut (transstadial transmission). These ASVs, despite being a small number, constituted a considerable proportion of the microbiota of each mosquito developmental stage (30–49% of the sequences) and were prevalent in a high number of samples being part of the core microbiota of each stage. Therefore, our results seem to support the important role of transstadial transmission in the colonization of mosquito adult gut, as already demonstrated by Coon et al. (2014), and the fact that a considerable part of mosquito microbiota is acquired during the aquatic life stages through the aquatic larval habitats (Bascuñán et al., 2018; Guégan et al., 2018). For example, in this study genera *C39* (family *Rhodocyclaceae*), *Aurantimicrobium* (family *Microbacteriaceae*) and family *Burkholderiaceae* were the main contributions of water bacterial communities to the microbiota of mosquito immature stages (Supplementary Figure 8). *C39*, which was one of the main genera constituting water community, got established in larval microbiota, but tended to decrease in subsequent mosquito stages, while genus *Aurantimicrobium* and family *Burkholderiaceae* seemed to find favorable conditions in larval and pupal guts, with especially the latter being able to proliferate (Figure 4F and Supplementary Figure 8).

### From Larvae to Adults: Metamorphosis and Change in Diet

The idea that a relevant portion of mosquito microbiota is constituted by bacteria acquired transstadially suggests that the process of bacterial clearance, which was thought to eliminate most of the microbial communities associated with larval and pupal midgut during mosquito metamorphosis (Moll et al., 2001), is not complete, as already advocated by recent studies (Coon et al., 2014; Duguma et al., 2015; Guégan et al., 2018). This is further supported by the fact that we did not observe a consistent decrease in bacterial diversity from larval to pupal to adult stages (Figure 1 and Supplementary Figure 4). Nevertheless, only 36% of the ASVs detected in larvae were detected in pupae and 24% of those observed in pupae were found in adults (Figure 3). Therefore, a considerable portion of ASVs from the previous stages were lost during mosquito development, and this could have happened during the renewal of the intestinal epithelial layers occurring during mosquito metamorphosis. Consequently, mosquito metamorphosis, even without completely eliminating gut microbiota, plays a role in the differentiation of bacterial communities throughout mosquito life cycle (Figure 2). The most evident change was the turnover in dominance between *Gammaprotebacteria* and *Alphaproteobacteria* from the immature to adult mosquito stage (Supplementary Figure 7). It is likely that this change is also a consequence of the switch in diet that mosquitoes experience reaching the adult phase. During their life mosquitoes shift from aquatic habitats, where larvae and pupae develop, mainly consuming microorganisms and small invertebrates, to terrestrial habitats, where adults feeds on extrafloral nectaries, with females also usually blood feeding on vertebrates. The change in diet can impact on mosquito microbiota in different ways. On one side, it may trigger the proliferation of some bacteria and the simultaneous decline of others: the release of hydrolases during digestion, the oxidative stress caused by a blood meal and the fact that the different food sources, based on their composition, may, or may not, provide fundamental nutrients for the growth of certain bacteria, represent selective pressures for bacteria inhabiting mosquito gut (Minard et al., 2013; van Tol and Dimopoulos, 2016). For example, the genus *Asaia* (family *Acetobacteraceae*), was detected at low percentages in water and mosquito immature stages but dominated the bacterial communities of the adult gut (50%). Since *Asaia* is often detected in flower nectar (Yamada et al., 2000; Tanasupawat et al., 2009), it might have been acquired by adult mosquitoes from the environment through nectar feeding. Nevertheless, the fact that in our study *Asaia* was represented by a single ASV, detected at high prevalence in all the sample types (WLPA and core ASV) (Figure 5), increases the chances that this bacterium was transmitted transstadially from the larval breeding sites to mosquito adult gut, where it found conditions favorable for its proliferation. Indeed, due to a mainly sugar-based diet, the adult mosquito gut is a sugar-rich environment, which *Acetobacteraceae* are know to be adapted to (Crotti et al., 2010). At the same time, most of the bacteria that constituted pupal microbiota and were transferred to the adult stage, such as *Burkholderiaceae*, *Aeromonas*, *C39*, *Flavobacterium*, *Hydrogenophaga* and *Aurantimicrobium* (64% of pupal community), considerably decreased in abundance in adults (3%) (Figure 4F). Most of these are bacteria adapted to aquatic environments which most likely found advantageous conditions inside the gut of larvae and pupae, which conduct an aquatic life, but may particularly suffer from the shift to a terrestrial lifestyle in adults. On the other side, the shift in diet may cause the acquisition of new bacteria from the new nutritional sources: the 30% of the total ASVs detected in our study (77% of those detected in adults) which were unique to adult gut probably represent the contribution in bacteria from the new diet to adults. Most of these belonged to genera usually associated to adult mosquito nutritional sources: *Pseudomonas*, *Pantoea* and the family *Enterobacteriaceae* are among the most prevalent bacteria recovered from floral nectar or from plant surfaces (Junker et al., 2011; Álvarez-Pérez et al., 2012; Wang et al., 2012); *Gilliamella* is a dominant gut bacterium of adult honey bees and bumble bees involved in the metabolism of sugars (Zheng, 2016); *Dyella* has been reported as a plant endophyte (Contreras et al., 2016) (Figure 4E).

In our study male and female mosquitoes showed similar microbiota structure and composition (Supplementary Figures 5, 11), which is in contrast to previous findings (Zouache et al., 2011; Valiente Moro et al., 2013). This may be explained by the fact that in this study we have used only females that were not engorged with blood, even though we cannot establish whether they had prior access to a blood or a sugar meal. In our data, though, there were no evident effects of recent blood meals in females. For instance, males and females did not show significant differences in bacterial diversity, whereas blood meals are known to reduce community diversity (Wang et al., 2011). Moreover, the microbiota of both males and females was dominated by *Asaia*, with *Pseudomonas* as second main component, and high abundances of *Acetobacteraceae* and *Pseudomonas* are known to be associated to sugar-fed mosquitoes (Muturi et al., 2018a). Together these evidences seem to indicate the possibility that the majority of females that were analyzed in this study had acquired a sugar meal, which may explain why we did not observed significant differences between male and female microbiota in bacterial diversity and community structure.

### Bacterial Candidates for Microbiota-Based Control Measures

Understanding how the microbiota of *Ae. koreicus* changes through its life cycle allows the selection of bacterial candidates that could be used in microbiota-based control strategies against this mosquito vector and its pathogens, i.e., Japanese encephalitis virus, chikungunya virus and filarial nematodes (Miles, 1964; Shestakov and Mikheeva, 1966; KCDC, 2007; Montarsi et al., 2015a; Ciocchetta et al., 2018).

Our results confirm in *Ae. koreicus* most of the properties of *Asaia* already reported from other mosquito species which makes this bacterial symbiont one of the most promising candidates for paratransgenesis (Favia et al., 2007; Bongio and Lampe, 2015; Mancini et al., 2016). We show that *Asaia* is dominant (it represents 50% of adult mosquito bacteria), highly prevalent (it is part of the core microbiome of each developmental stage) and widely distributed in *Ae. koreicus* body, similarly to what reported in *Anopheles stephensi*, *Anopheles gambiae* and *Ae. aegypti* (Favia et al., 2007; Crotti et al., 2009; Chouaia et al., 2010; Mancini et al., 2016). The fact that *Asaia* was detected in *Ae. koreicus* gut means that it infests a part of the mosquito body where it can be in close contact with the pathogens potentially transmitted by this species, such as arboviruses and filarial nematodes, and where it can be used to express antipathogen effector molecules. In addition, the dominance and prevalence of *Asaia* seem to suggest its presence also in the reproductive organs of *Ae. koreicus*, and, therefore, vertical transmission, which would favor the dissemination of the engineered bacteria in vector populations and decrease the need of repeated release experiments (Favia et al., 2007, 2008; Damiani et al., 2010). Then, the transstadial transmission of *Asaia* suggested by our results would enable the transformed bacteria to be introduced into wild populations at the larval stage, and then be naturally transferred to the adults (Chavshin et al., 2015). Finally, *Asaia* can be easily cultured and genetically manipulated to produce effector molecules, as demonstrated in an experiment aimed at inhibiting *Plasmodium berghei* development in *An. stephensi* (Bongio and Lampe, 2015). Our results further expand the group of mosquito species which *Asaia* is stably associated with (*An. gambiae*, *An. stephensi*, *Anopheles maculipennis*, *Ae. albopictus*, *Ae. aegypti*, *Culex pipiens*, *Culex quinquefasciatus*, *Culex nigripalpus*) (Favia et al., 2007; Crotti et al., 2009; Damiani et al., 2010; Gusmao et al., 2010; Minard et al., 2013; Mitraka et al., 2013; De Freece et al., 2014; Duguma et al., 2019), demonstrate the possibility to develop a paratransgenic system based on *Asaia* in *Ae. koreicus* and, therefore, support the use of *Asaia* as an universal delivery vehicle for the paratransgenic control of mosquito-borne diseases.

At the same time, the presence of *Asaia* in *Ae. koreicus* seems to impede the infection with *Wolbachia*, which indeed was not detected in any *Ae. koreicus* developmental stage, confirming the previous finding from adults (Rosso et al., 2018). This could be caused by the fact that *Asaia* interferes with *Wolbachia* vertical transmission through a competition for reproductive organs colonization, as discovered in *Anopheles* mosquitoes (Hughes et al., 2014; Rossi et al., 2015), and, therefore, argue against the implementation of *Wolbachia*-mediated control strategies in *Ae. koreicus.*

Several other bacteria have been considered for paratransgenesis based on their properties and distribution in mosquito vectors. For instance, *Thorsellia*, in particular *T. anophelis*, has been proposed for its dominance and persistence in life stages of a wide range of mosquitoes (Lindh et al., 2005; Rani et al., 2009; Wang et al., 2011; Chavshin et al., 2014). Our study represents the first record of *T. anophelis* in *Aedes* mosquitoes. In particular, we found *T. anophelis* as the second most abundant genus in *Ae. koreicus* larvae (10%), which is in line with the dominant role of this bacterial species in *Culex* immature stages (Duguma et al., 2015, 2017), and also in water, pupae and adults, even if at very low abundances (<1%). The fact that we found *T. anophelis* ASVs shared among multiple sample types (WLP or LPA), even if none was shared across all (WLPA), seems to confirm its transstadial transmission properties, as already suggested by its detection in all developmental stages of *Culex* species (Duguma et al., 2015, 2017) and from both adults and breeding site water of *An. gambiae* (Briones et al., 2008). Anyway, the main limitation of *Thorsellia* is the fact that it has not yet been demonstrated to be transformable to express effectors against mosquito pathogens.

*Pseudomonas* has also been proposed as paratransgenetic symbiotic control agents. We detected *Pseudomonas* as the second most abundant bacterial genus in adults of *Ae. koreicus*, although it reached only 4% abundance, since adult microbiota is largely dominated by *Asaia* (50%). Conversely, a previous study on *Ae. koreicus* adult microbiota reported *Pseudomonas* as the most dominant genus at over 60% abundance, followed by *Asaia* at 14% (Rosso et al., 2018). Even if with different proportions, both studies demonstrate that *Asaia* and *Pseudomonas* are the main constituents of adult *Ae. koreicus* gut microbiota. Consistently, *Pseudomonas* has been found also as a common member of the gut microbiota of malaria mosquitoes (Chavshin et al., 2012, 2014, 2015), in *Ae. albopictus* (Rosso et al., 2018) and *Ae. aegypti* (David et al., 2016), and in several *Culex* and *Anopheles* species in Belgium (Raharimalala et al., 2016). In our study, *Pseudomonas* was also detected in immature stages and water but at lower abundance than in adults, and as part of the core microbiome of both pupae and adults. Three *Pseudomonas* ASVs were shared across all sample types (WLPA), suggesting that *Pseudomonas*, besides being acquired by adults by nectar feeding as described above, is also potentially acquired by *Ae. koreicus* larvae from the aquatic environment and transstadially transmitted to the adult stage. This is in line with what already demonstrated in *An. stephensi* (Chavshin et al., 2015). The fact that *Pseudomonas* seems to escape from bacterial removal during metamorphosis through the colonization of the Malpighian tubules (Chavshin et al., 2015) opens up the possibility to use *Pseudomonas* for paratransgenesis against pathogens spending part of their life cycle in these organs, which is the case of *Dirofilaria immitis* in *Ae. koreicus* (Montarsi et al., 2015a). In addition, *Pseudomonas* can be cultured and is suitable for genetic transformation (Choi et al., 2006). Therefore, *Pseudomonas* represents a promising candidate for paratransgenesis, although the pathogenicity of any candidate species needs to be assessed first, since some are known to cause diseases in plants and animals, including humans (He et al., 2004; Morris et al., 2008).

## Conclusion

Our study demonstrates the importance of studying mosquito microbiota composition and the factors influencing its variation throughout host life cycle in order to develop microbial intervention measures to limit vector competence. In particular, transstadially transmitted bacteria represent interesting candidates to apply paratransgenetic approaches, thanks to their persistence and ease of introduction in vector populations. Dominance, stable association with one or more developmental stages, and localization within vector body are also key features. Our study revealed that, if on one side the use of *Wolbachia* in *Ae. koreicus* is hindered by the presence of *Asaia*, on the other the bacterial genera *Asaia*, *Pseudomonas* and *Thorsellia* seem to be promising candidates for paratransgenesis in this species. Future studies are needed to analyze the microbiota of adult individuals in other mosquito body regions, such as reproductive organs, which are important to confirm vertical transmission properties of the bacteria, and salivary glands, which play a key role in pathogen transmission. To date paratransgenesis has been explored mainly in *Anopheles* mosquitoes for the control of malaria, thus future efforts should focus primarily on assessing paratransgenesis approaches in *Aedes* and *Culex* mosquitoes and on characterizing efficient arbovirus-specific antiviral molecules which, as yet, have not been identified.

## Data Availability Statement

The dataset generated and analyzed during this study is available in the NCBI Sequence Read Archive (SRA) repository under the accession number PRJNA549354. The *Asaia* 16S rRNA gene fragment sequence generated by PCR in this study to confirm the taxonomic assignment of the correspondent ASV was deposited in GenBank under the accession number MN099438.

## Author Contributions

VT, FR, and AR designed the study. DA and VT collected the samples in the field. FR performed the laboratory experiments. NA analyzed the data and wrote the manuscript. MM helped in the statistical analyses. MP prepared the amplicon library and the sequencing database. NA, VT, FR, MM, DA, and AR discussed the results and reviewed the manuscript. All authors read and approved the final manuscript.

## Conflict of Interest

The authors declare that the research was conducted in the absence of any commercial or financial relationships that could be construed as a potential conflict of interest.

## Footnotes

1 https://qiime2.org/

2 https://www.arb-silva.de

3 https://www.arb-silva.de
