# Supplementary material for: Changes in Microbiota Across Developmental Stages of Aedes koreicus, an Invasive Mosquito Vector in Europe: Indications for Microbiota-Based Control Strategies
Source: Front Microbiol. 2019 Dec 10;10:2832. doi: 10.3389/fmicb.2019.02832 (PMC6914824; doi:10.3389/fmicb.2019.02832)
Supplement: Supplementary file 1 [file Data_Sheet_1.PDF]

SUPPLEMENTARY FIGURES

Supplementary Figure 1

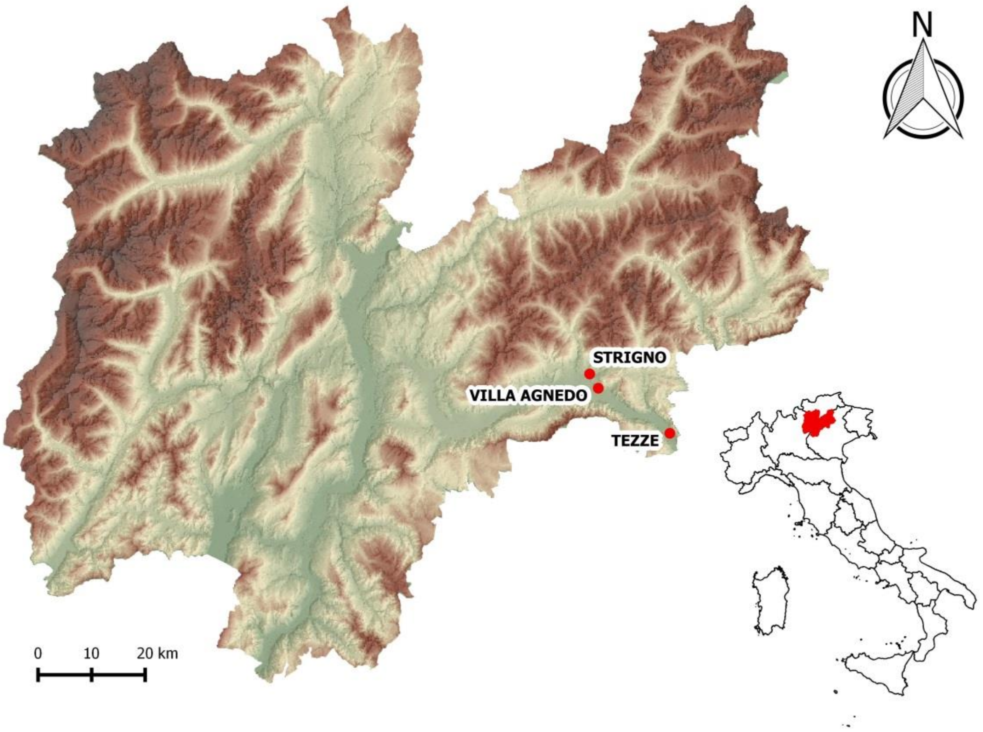

Suppl. Fig. 1. Map of the study area (Province of Trento, north-eastern Italy) and of the 3 sampling sites.

Supplementary Figure 2

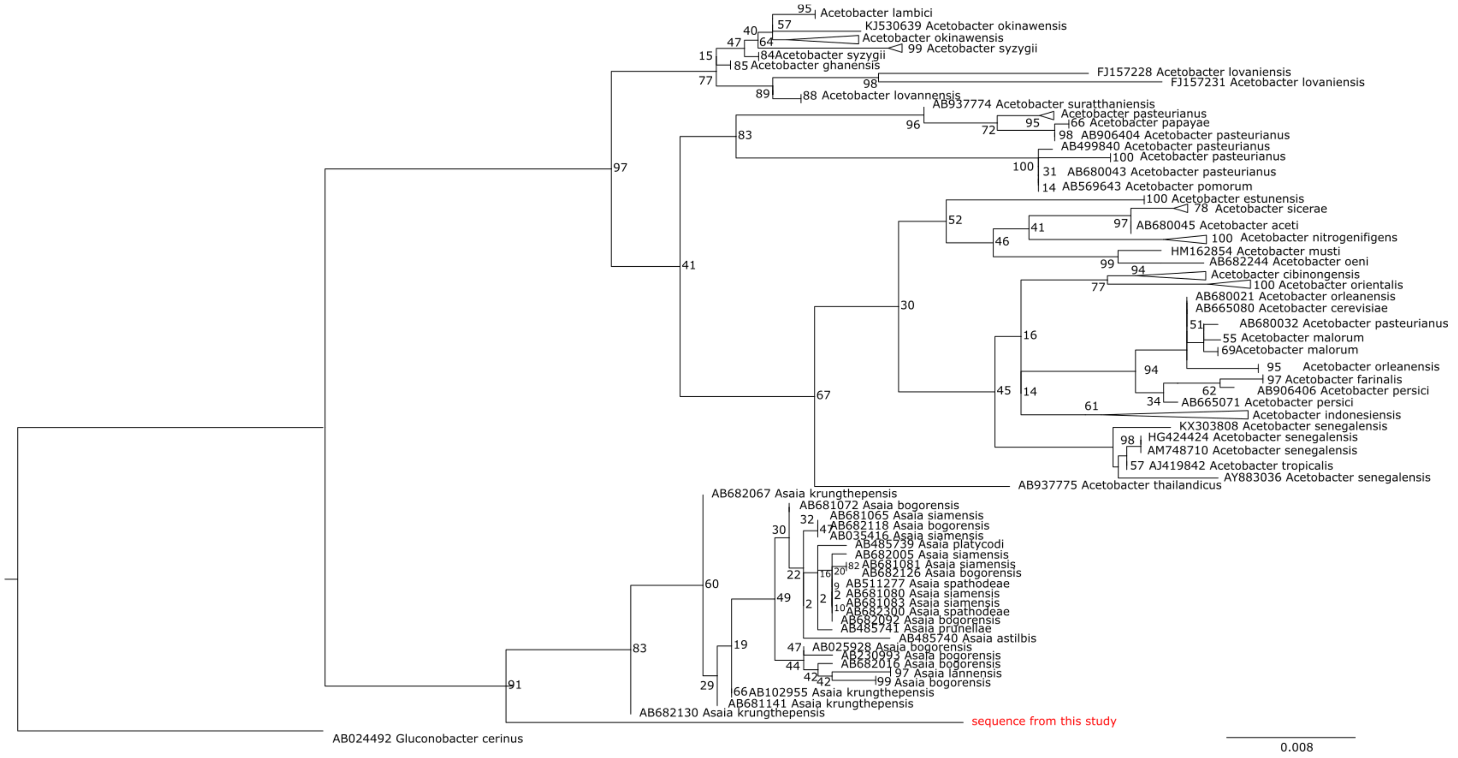

Suppl. Fig. 2. Phylogenetic analysis of the long *Acetobacteraceae* sequence generated by PCR together with *Acetobacter* and *Asaia* sequences from the Silva database. Maximum-likelihood phylogenetic tree inferred using 1500 bp of the 16S rRNA gene. Node robustness was assessed with 500 rapid bootstrap pseudoreplicates. Numbers next to the internode branches indicate bootstrap support. The sequence generated in this study is highlighted in red. The scale bar indicates 0.008 nucleotide substitutions per site.

Supplementary Figure 3

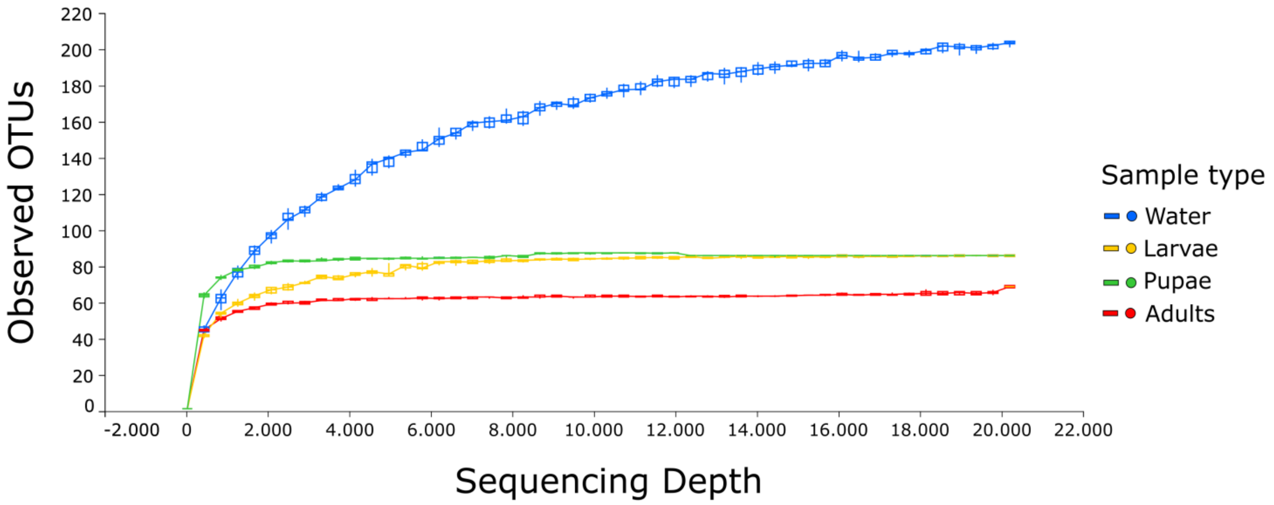

Suppl. Fig. 3. Rarefaction analysis for each sample type. Rarefaction curves obtained from the water, larvae, pupae and adults samples showing the number of unique ASVs (observed OTUs metric) as a function of sequencing depth for each sample type. The curves were calculated at a maximum rarefaction depth of 20,194 sequences/sample.

Supplementary Figure 4

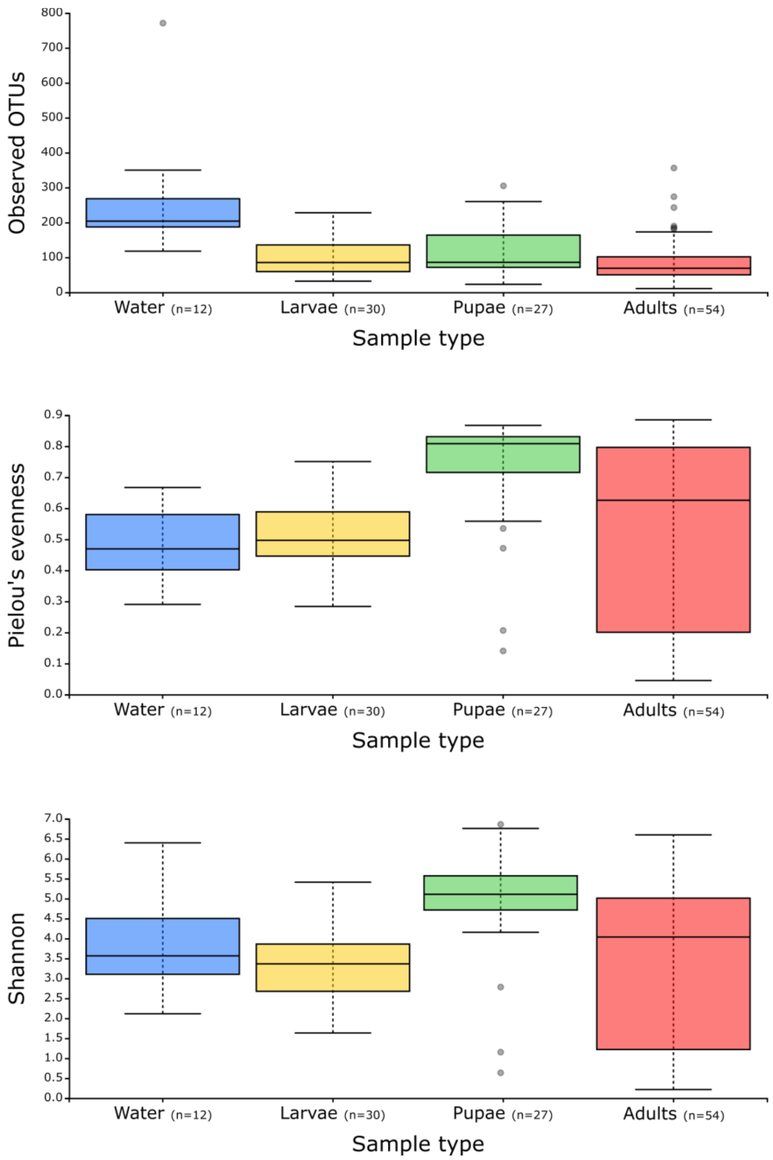

Suppl. Fig. 4. Alpha diversity metrics for each sample type. Box plot showing alpha diversity measures for each sample type. Each metric was calculated at a rarefaction depth of 20,194 sequences/sample. Boxes represent the interquartile range (IQR) between the first and third quartiles (25th and 75th percentiles, respectively), and the horizontal line inside the box defines the median. Whiskers represent the lowest and highest values within 1.5 times the IQR from the first and third quartiles, respectively. Circle symbols indicate outliers (values greater than 1.5 times and less than three times the IQR).

Supplementary Figure 5

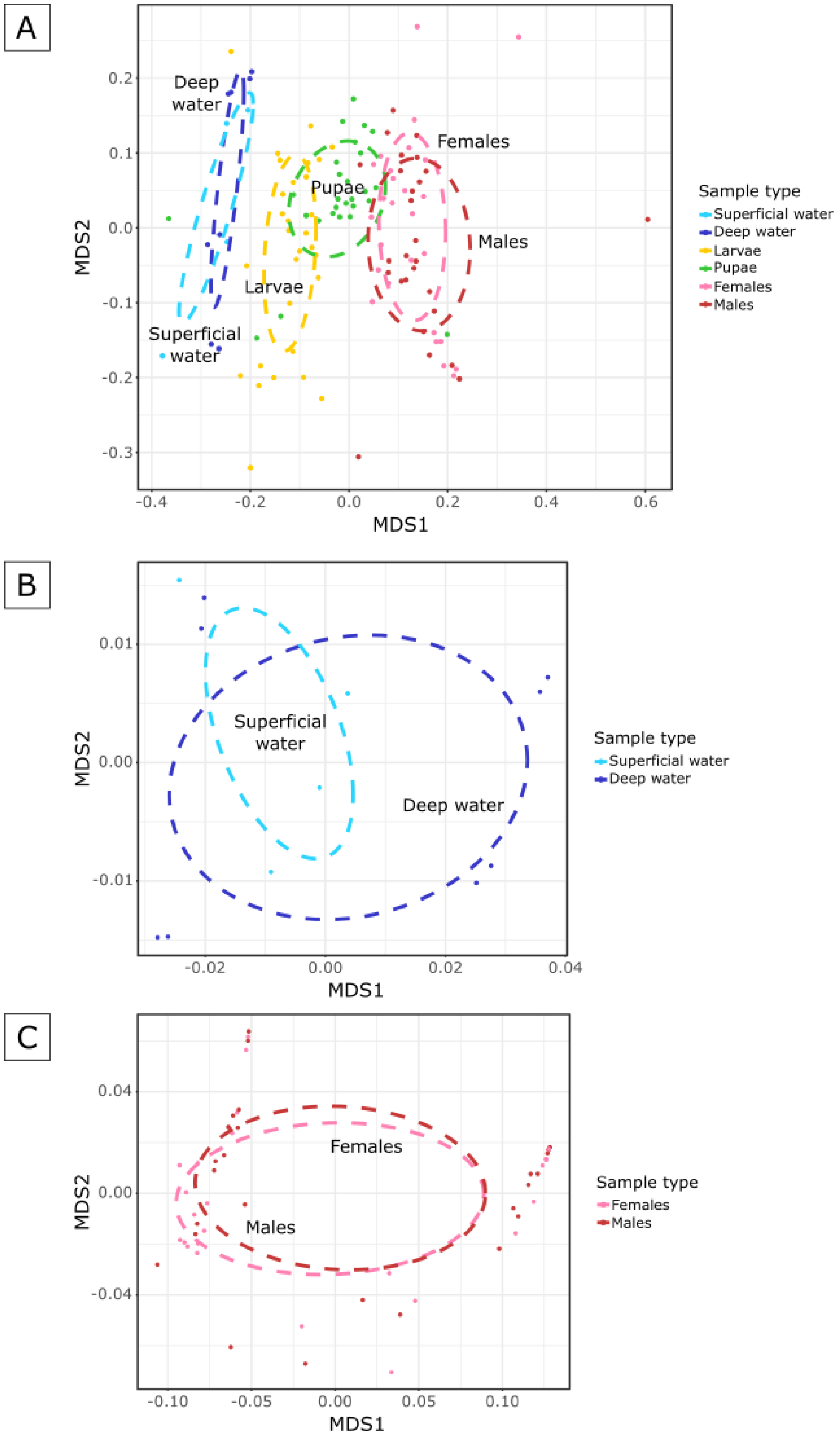

Suppl. Fig. 5. Similar bacterial community structure between surface and deep water samples and between adult males and females. Non-metric multidimensional scaling (NMDS) analysis of Bray-Curtis distances between all sample types (A), only surface and deep water samples (B) and only male and female adult samples (C).

Supplementary Figure 6

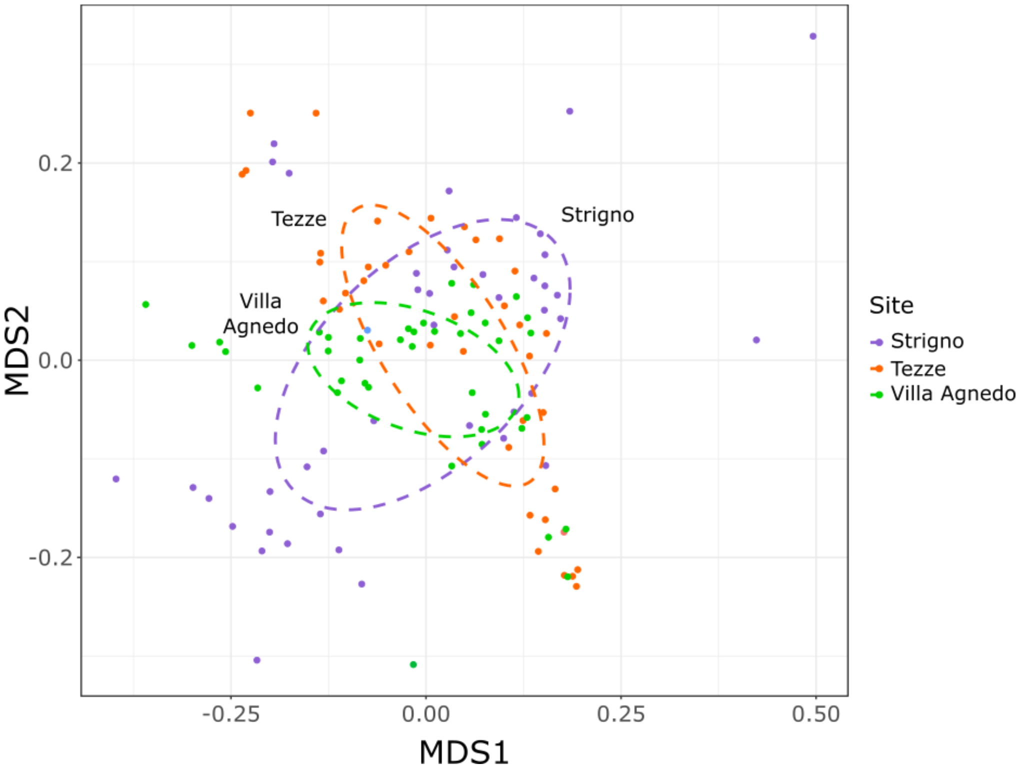

Suppl. Fig. 6. Absence of distinct clustering pattern between bacterial communities based on sampling sites. Non-metric multidimensional scaling (NMDS) ordination of Bray-Curtis distances between the microbial communities of the three sampling sites.

Supplementary Figure 7

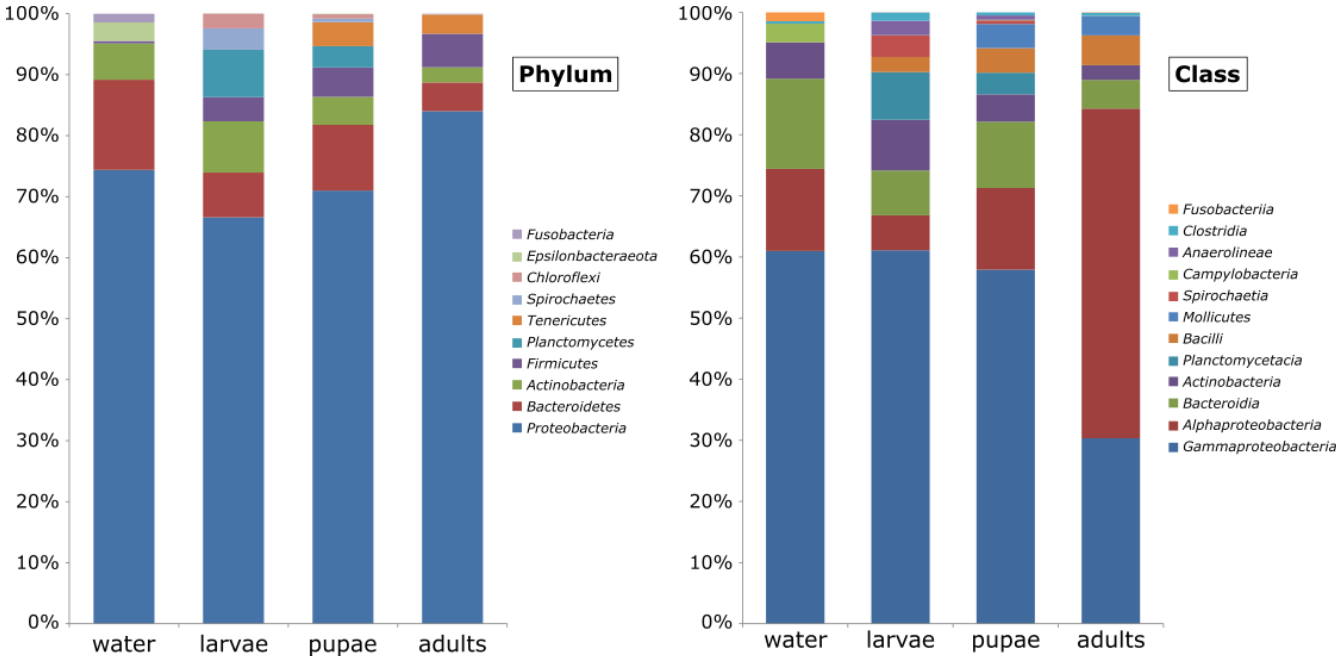

Suppl. Fig. 7. Taxonomic composition of the bacterial communities of each sample type. Bar chart representations of the relative abundances of the bacterial phyla and classes found in each sample type.

Supplementary Figure 8

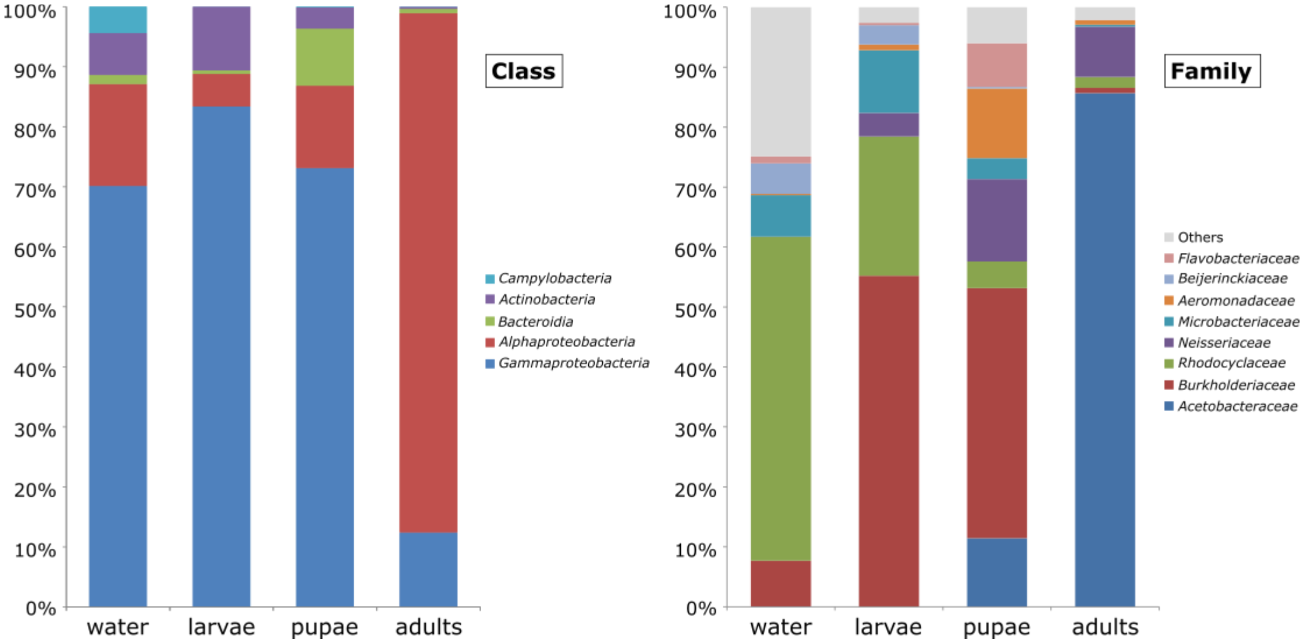

Suppl. Fig. 8. Taxonomic profile at class and family level of the ASVs shared between all sample types (WLPA).

Supplementary Figure 9

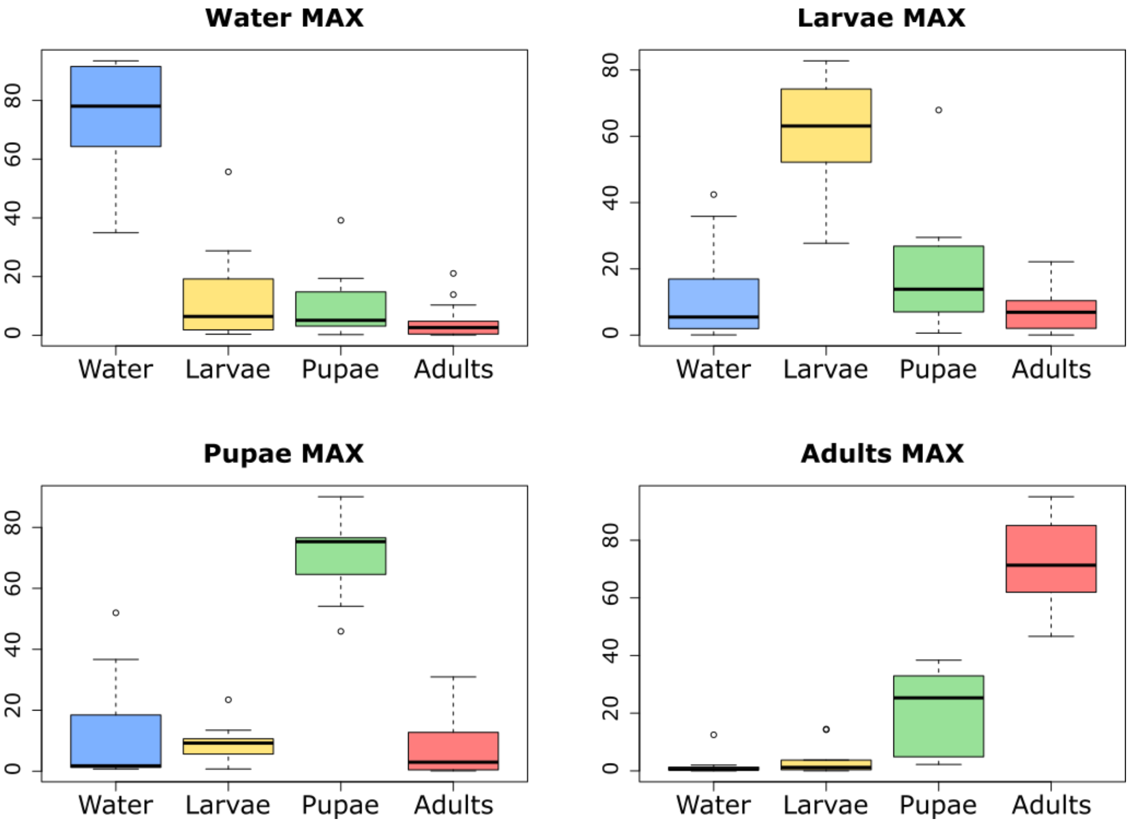

Suppl. Fig. 9. Percent abundance per sample of the shared ASVs (WLPA) reaching their maximal abundances in each of the four sample types.

Supplementary Figure 10

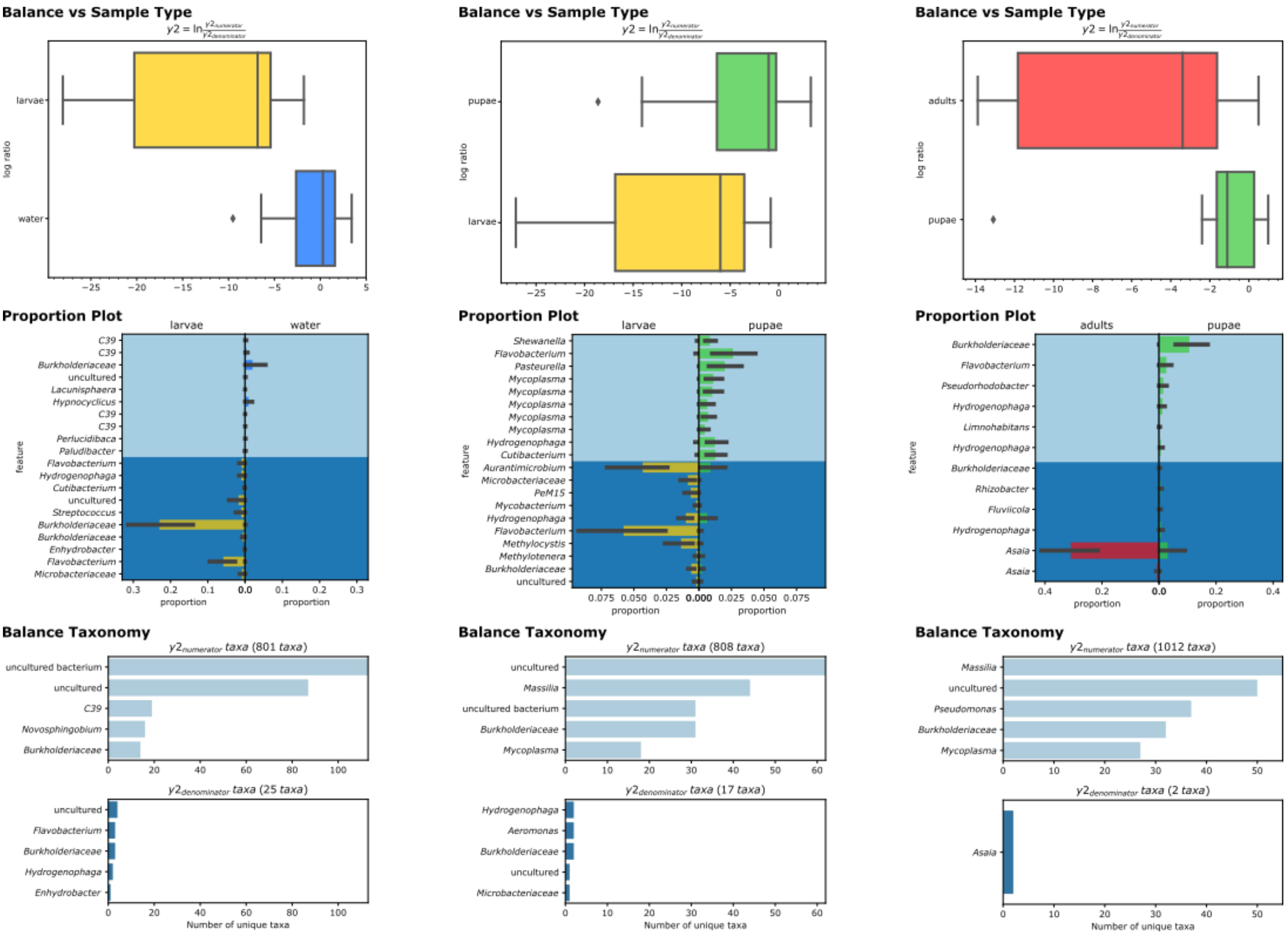

Suppl. Fig. 10. Principal balances quantifying the microbial community shifts between pairs of sample types. Top: box plots showing the distribution of log ratios of ASVs sorted at genus level for balance y2 between the two sample types compared. Middle: proportion plots based on balance tree calculation showing shifts in major genera between each pair of sample types. Bottom: bar diagrams showing the number of major bacterial taxa at the genus level associated to each sample type that contributed to shifts in y2 balance. Identification at the family level is reported when genus identification is not available.

Supplementary Figure 11

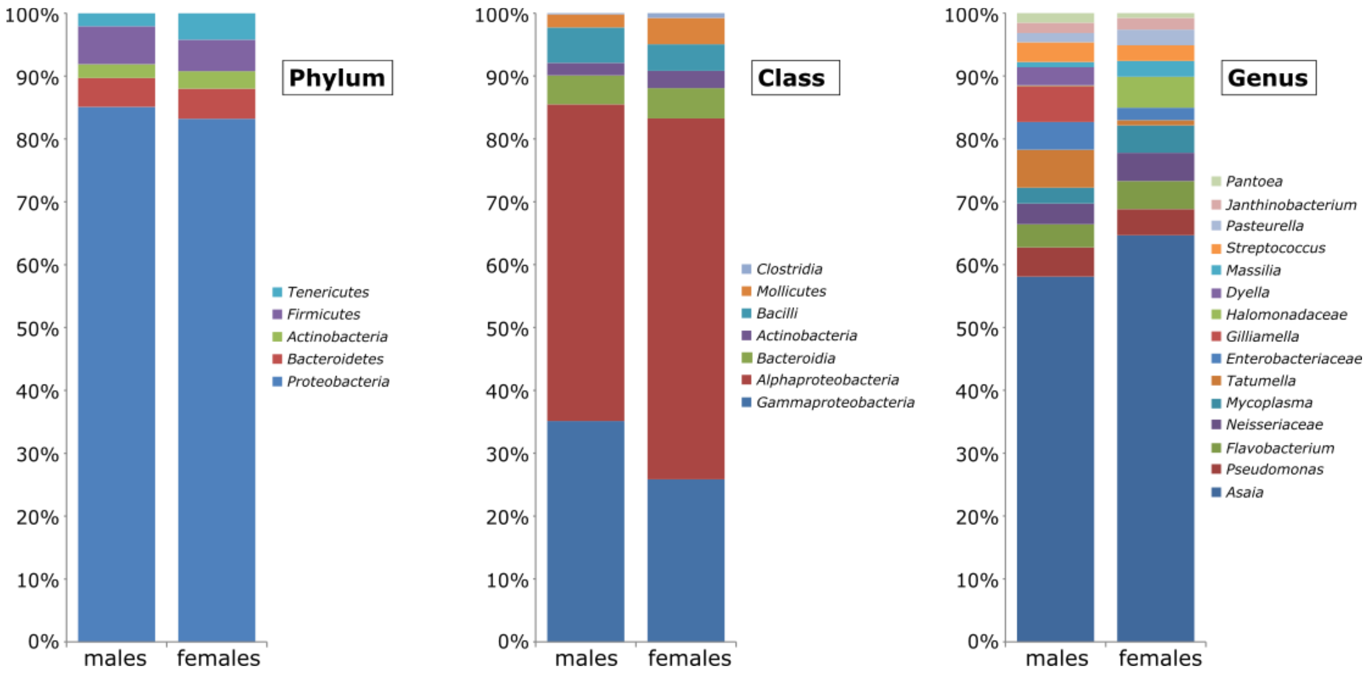

Suppl. Fig. 11. Taxonomic profile at phylum, class and genus level of the adult male and adult female bacterial communties.

SUPPLEMENTARY TABLES

Suppl. Tab. 1. Number of reads per sample obtained at each step of the bioinformatics pipeline. Number of reads for each sample generated by Illumina sequencing, obtained after each step of the DADA2 pipeline (quality filtering, de-noising, merging, chimeric sequences removal) and after removal of contaminants, together with basic statistics (total, average and standard deviation).

Suppl. Tab. 2. Statistical comparison of alpha diversity values between sample types. The effects of sample type on four alpha diversity metrics was evaluated using both an "all-group" and a pairwise Kruskal-Wallis test. A Benjamini & Hochberg FDR corrected p-value ("q-value") is reported. The pairs with a significant value (*: q-value < 0.01) are highlighted in bold. Here, surface and deep water samples are merged and presented as "water" samples. Similarly, adult female and adult male samples are merged and presented as "adult" samples.

Suppl. Tab. 3. Statistical comparison of alpha diversity values between sample types. The effects of sample type on four alpha diversity metrics was evaluated using both an "all-group" and a pairwise Kruskal-Wallis test. A Benjamini & Hochberg FDR corrected p-value ("q-value") is reported. The pairs with a significant value (*: q-value < 0.01) are highlighted in bold. Here, both surface and deep water samples, and adult female and adult male samples are separated.

Suppl. Tab. 4. Taxonomic composition of the bacterial communities of each sample type at different taxonomic levels. ASVs relative abundances at phylum, class, order, family and genus level in the water, larvae, pupae and adults microbiota. ASVs with an abundance < 0.01% of the total read count were removed. Only groups with a maximal abundance >1% in one of the four sample types are shown to simplify the visualization of the results, except for genera where the threshold was set to 0.5%.

Suppl. Tab. 5. ANCOM results. Differential abundance analysis using ANCOM between each pair of sample types (water-larvae; larvae-pupae; pupae-adults). The W value represents the number of taxa that a given taxa is tested to be significantly different against. The abundances of the taxa identified by ANCOM were inspected in each sample. After inspection, sparse taxa presenting zero abundance in most samples in the feature table, and therefore giving false-positive results with ANCOM, were not confirmed to be differentially abundant.

Suppl. Tab. 6. Taxonomic composition of the bacterial communities at different taxonomic levels, with communities of adults divided into males and females. ASVs relative abundances at phylum, class, order, family and genus level in the microbiota of water, larvae, pupae, adult males and adult females. ASVs with an abundance < 0.01% of the total read count were removed. Only groups with a maximal abundance in one of the four sample types >1% are shown to simplify the visualization of the results, except for genera where the threshold was set to 0.5%.
